# Positional Cloning of the Flowering Time QTL *qFT12-1* Reveals the Link Between the Clock Related *PRR* Homolog With Photoperiodic Response in Soybeans

**DOI:** 10.3389/fpls.2019.01303

**Published:** 2019-10-15

**Authors:** Yuqiu Li, Yingshan Dong, Hongyan Wu, Bo Hu, Hong Zhai, Jiayin Yang, Zhengjun Xia

**Affiliations:** ^1^Key Laboratory of Soybean Molecular Design Breeding, Northeast Institute of Geography and Agroecology, The Innovative Academy of Seed Design, Chinese Academy of Sciences, Harbin, China; ^2^University of Chinese Academy of Sciences, Beijing, China; ^3^Soybean Research Institute, Jilin Academy Agricultural of Science, Changchun, China; ^4^Crop Development Center, Huaiyin Institute of Agricultural Sciences in Xuhuai Region of Jiangsu Province, Huaian, China

**Keywords:** soybean, flowering time, fine mapping, pseudo-response regulator, photoperiodic response

## Abstract

Flowering time and maturity are important agronomic traits for soybean cultivars to adapt to different latitudes and achieve maximal yield. Genetic studies on genes and quantitative trait loci (QTL) that control flowering time and maturity are extensive. In particular, the molecular bases of *E1-E4*, *E6*, *E9*, *E10*, and *J* have been deciphered. For a better understanding of regulation of flowering time gene networks, we need to understand if more molecular factors carrying different biological functions are also involved in the regulation of flowering time in soybeans. We developed a population derived from a cross between a landrace Jilincailihua (male) and a Chinese cultivar Chongnong16 (female). Both parents carry the same genotypes of *E1e2E3HaE4* at *E1*, *E2*, *E3*, and *E4* loci. Nighty-six individuals of the F_2_ population were genotyped with Illumina SoySNP8k iSelect BeadChip. A total of 2,407 polymorphic single nucleotide polymorphism (SNP) markers were used to construct a genetic linkage map. One major QTL, *qFT12-1*, was mapped to an approximately 567-kB region on chromosome 12. Genotyping and phenotyping of recombinant plant whose recombination events were occurring within the QTL region allowed us to narrow down the QTL region to 56.4 kB, in which four genes were annotated. Allelism and association analysis indicated *Glyma.12G073900*, a *PRR7* homolog, is the strongest candidate gene for *qFT12-1*. The findings of this study disclosed the possible involvement of circadian clock gene in flowering time regulation of soybeans.

## Introduction

Soybeans are an important crop globally and provide quality protein and oil for human and animal consumption (Graham and Vance, 2003). Flowering time and maturity are essential traits in crop cultivation. Soybeans, a typical short-day plant, are highly sensitive to day length. Molecular bases of flowering and maturity genes are important for understanding the mechanism regulating photoperiodic response and adaptation in soybeans (Zhao et al., 2016).

Flowering time and maturity regulated by photoperiod sensitivity genes or loci (Garner and Allard, 1927; Cober et al., 2014) have been reported for soybeans, *E1*, *E2* (Bernard, 1971), *E3* (Buzzell, 1971), *E4* (Buzzell and Voldeng, 1980), *E5* (McBlain and Bernard, 1987), *E6* (Bonato and Vello, 1999), *E7* (Cober and Voldeng, 2001), *E8* (Cober et al., 2010), *E9* (Kong et al., 2014), *E10* (Samanfar et al., 2017), and *J* (Ray et al., 1995). Moreover, many other quantitative trait loci (QTLs) have been mapped for flowering time and maturity using different populations (Tasma et al., 2001; Chapman et al., 2003; Watanabe et al., 2004; Funatsuki et al., 2005; Pooprompan et al., 2006; Komatsu et al., 2007; Khan et al., 2008; Liu and Abe, 2009; Cheng et al., 2011; Yamaguchi et al., 2014). A total of 293 QTLs on flowering time and maturity, including 104 QTLs of first flower, 178 QTLs of pod maturity (R8), 5 QTLs for pod maturity beginning (R7), and 6 QTLs for pod beginning (R3), have been documented in the public repository, Soybase (http://soybase.org/). Particularly, seven QTLs for flowering time (R1) were mapped to a rather large region of 28.2–45 cm on chromosome 12(LG H) (Kuroda et al., 2013; Lu et al., 2016; Fang et al., 2017; Mao et al., 2017; Liu et al., 2018); however, the molecular identities of these QTLs are still unknown.

In *Arabidopsis*, the five major flowering time pathways, e.g., the photoperiod, vernalization, gibberellin, autonomy, and aging, are well characterized. In the photoperiodic pathway, the photoperiodic flowering model of *GIGANTEA* *(GI)-CONSTANS (CO)-FLOWERING LOCUS T (FT)* is widely conserved among different plant species. The *GI* protein is a nuclear protein that links the circadian clock and the flowering time pathway. *GI* interacting with the Flavin-Binding, Kelch Repeat, F-Box 1 (FKF1) protein can induce *CO* and *FT* expression (Shim et al., 2017). In addition, *GI* can directly or indirectly regulate FT expression by interacting with *FT* repressors or binding to *FT* promoter regions in a way independent of *CO* (Sawa and Kay, 2011). *PSEUDO RESPONSE REGULATORS 3, 5, 7*, and *9* (*PRR3*, *PRR5*, *PRR7*, and *PRR9*), the additional members of the *TOC1* family, are core genes participating in additional loops essential for clock function (Mizuno and Nakamichi, 2005). In the soybean genome, PRR family seems to have undergone a different gene expansion process during evolution (Quecini et al., 2007).

The major flowering time gene, *E1*, encodes a legume specific transcription factor containing a B3-like domain that represses the expression of two florigen genes, *GmFT2a* and *GmFT5a*. Four main *E1* alleles, e.g., *E1*, *e1-as*, *e1-fs*, and *e1-nl*, have been identified in soybean cultivars. *E1* is the strongest genotype in suppressing the expression of *GmFT2a* and *GmFT5a*. The *e1-as* is a leaky allele that can suppress the expression of *GmFT2a* and *GmFT5a*, whereas both *e1-fs* and *e1-nl* are non-functional (Xia et al., 2012a). *E3* and *E4* encode the photoreceptors *PHYA3* (Watanabe et al., 2009) and *PHYA2* (Liu et al., 2008), respectively, which are involved in the regulation of *E1* (Xia et al., 2012a). Intriguingly, the soybean *E2* gene was identified to be a GIGANTEA homolog (*GmGIa*), there are three major haplotypes of *E2* (Watanabe et al., 2011).

Various allelic combinations at the *E1*, *E2*, *E3*, and *E4* loci controlling the absence of or reduced photoperiod sensitivity determine the diversification of soybean maturity and adaptation at different latitudes (Xu et al., 2013; Tsubokura et al., 2013a; Tsubokura et al., 2013b; Zhai et al., 2014; Jiang et al., 2014; Kurasch et al., 2017; Langewisch et al., 2017; Li et al., 2017; Gupta et al., 2017). These genes or loci also influence the yield, adaptability, and quality of soybeans (Xia et al., 2012b; Sayama et al., 2010; Guo et al., 2015). Diversified functions of CO homologs in soybeans have been disclosed; however, these finding lacks the support of QTL study. Intriguingly, soybean *E2* gene was identified to be a GIGANTEA homolog (*GmGIa*). Even now, we still cannot answer to what extent the GI-CO-FT module is conserved in soybean.

In 2018, a QTL for flowering time, *qFT12.1*, was mapped to a 2,703-kB region interval between the markers BARCSOYSSR_12_0220 and BARCSOYSSR_12_0368 on chromosome 12 (Liu et al., 2018). Although the studies indicated functional genes exist in this region that controls flowering time, it has not been deciphered at high resolution. In this study, a major QTL for flowering time was detected on chromosome 12 in a mapping population derived from a cross between a Chinese landrace and an elite cultivar. Subsequently, fine mapping enabled us to delimit this region to 56.4 kB. Based on allelism and association analysis, *Glyma.12G073900*, a homolog of the clock gene *PRR7*, is the strongest candidate gene for *qFT12-1*, the flowering time QTL. This finding showed the possible involvement of circadian clock gene in flowering time regulation in soybeans.

## Materials and Methods

### Plant Material and Phenotyping

An F_2_ population of 322 individuals derived from a cross between Jilincailihua (JLCLH) and Changnong 16(CN16) was used for QTL mapping. Both parents are carrying the same genotypes of *E1e2E3HaE4* at *E1*, *E2*, *E3*, and *E4* loci. Female parent CN16 (*E1e2E3HaE4*), an elite cultivar that has been widely cultivated in the Northern China, was bred in 2003 at the Changchun Academy of Agricultural Sciences, China. The male parent, JLCLH (*E1e2E3HaE4*), was a Chinese landrace with a relatively later flowering phenotype. Phenotypic evaluation was conducted at two geographic locations: HARBIN, the campus of Northeast Institute of Geography and Agroecology, Harbin, Heilongjiang (45°70’ N, 126° 64’ E) and HAILUN, Hailun experiment station of Northeast Institute of Geography and Agroecology, Hailun, Heilongjiang (47° 47N, 126° 97’E). Flowering time (R1) of each plant was defined as days from emergence (DAE) to the opening of the first flower (Fehr et al., 1971). Plots were 5-m long with 0.6-m row spaces. Plants were generally 20 cm apart. We followed standard local agricultural practices to control insects and weeds. Plants were individually harvested.

In 2015, the F_2_ population was grown at HARBIN with 178 plants and at HAILUN with 135 plants. Subsequently, subpopulations were phenotyped from 2015 to 2018 at both HARBIN and HAILUN. In 2016, we planted 44 F_2:3_ lines whose parent plants had recombination at the QTL region between markers to confirm the authenticity of the QTL detected and to evaluate the suitability for fine mapping the QTL. Based on the analysis of field performance, we selected 4 F_2:3_ lines to develop subpopulations for the first round of fine mapping. We further identified seven recombinant plants whose progenies were phenotyped at HARBIN and HAILUN in 2017. Genotyping was performed using 18 markers within *qFT12-1* region (**Supplementary Table S1**). In 2018, the progenies of important recombinants harvested in 2017 were phenotyped for R1 to evaluate the stability of the phenotypic performance.

### DNA Isolation

DNA extraction from young leaves was carried out using the CTAB method with minor modifications (Xia et al., 2007). PCR was conducted in a total reaction volume of 10 μl containing 100-ng genomic DNA,10×PCR buffer (with 15 mM/L Mg^2+^), 0.2 μmol L^-1^ of each primer, 0.2 mmol L^-1^ dNTPs, 1 U Taq polymerase (Transgen Biotech, Beijing, China) in distilled water. PCR was performed using the following program: denaturation at 94°C for 5 min, 35 cycles of denaturation at 94°C for 30 s, annealing at 52°C for 30 s, extension at 72°C for 20 s, and a final extension at 72°C for 10 min before cooling down to 16°C.

### Genetic Linkage Map Construction and QTL Mapping

The F_2_ population was used to construct linkage maps. A total of 96 randomly selected individuals (grown at HAILUN in 2015), along with parents, were genotyped using Illumina SoySNP8k iSelect BeadChip (Yang et al., 2017). A total of 7,189 SNPs were specifically manufactured by Infinium HD Ultra. SNP genotyping was performed with the Illumina Iscan platform (Illumina, Inc., San Diego, CA). Genetic linkage analysis and map construction were performed with IciMapping_4.0 (Meng et al., 2015).

The recombination threshold value was set at 0.40 and the Kosambi mapping function was used to convert recombination frequencies into map distances. Markers showing distorted segregation were included in the linkage analysis.

QTL analysis was carried out using the software IciMapping version 4.0. The Inclusive Composite Interval Mapping (ICIM-ADD) model was used to detect QTLs. For the ICIM-ADD, a LOD score of 3.0 was used as a minimum to declare the significance of a QTL in a particular genomic region. One thousand permutations at a probability of 0.05 were also conducted to identify the genome-wide LOD (Meng et al., 2015). QTLs with an LOD score exceeding the genome-wide LOD were declared as significant QTLs, whereas QTLs with LOD less than the genome-wide LOD but more than 3.0 were identified as suggestive QTLs. MCIM was used to map QTLs with additive and epistatic effects. This analysis was performed using a 2D genome scan, with a 1-cM walking speed and 10-cM window size. Significant thresholds (critical F-values) for QTL detection were calculated with 1,000 permutations and a genome-wide error rate of 0.05.

Genotypic and phenotypic data were analyzed using the IBM SPSS Statistics 20 Program. The analysis of variance (ANOVA) of correlation was evaluated under the general linear model in SPSS. Flowering times were tested for deviations from normality using the parameters of kurtosis and skewness by SPSS SPSS 16.0 software (SPSS Inc., Chicago, IL, USA).

### Whole-Genome Resequencing of Parents and Marker Development

The parents were sequenced on Illumina HiSeq2000 platform (Illumina, Inc., San Diego, CA, United State), and 125-bp paired-end reads with insert sizes of roughly 300 bp were generated. Forward and reverse paired reads were split for each sample into two separate files (option –split-files). Reads were quality trimmed by NGS QC Toolkit (v2.3.3) (Patel and Jain, 2012) with default parameters. For each sample, only reads passing the quality filtering as matching pairs were retained and aligned to the reference genome Gmax_275_Wm82.a2.v1 using the Burrows-Wheeler Alignment Tool (BWA) v.0.6.2 (Liu and Abe, 2009). The resulting SAM files were converted to sorted BAM files compliant to the Genome Analysis Toolkit (GATK) format by Picard Tools v.1.77 (http://picard.sourceforge.net/) using the tools, in the following order, CleanSam, SamFormatConverter, and AddOrReplaceReadGroups. GATK-compliant BAM files were submitted to GATK v.2.3-3 for preprocessing procedures. The InDel variants above were used to develop Indel markers and SNP variants were used to develop CAPS/dCAPS or High-Resolution Melting (HRM) markers (**Supplementary Table S1**).

PCR products of InDel primers above were separated using 12% nondenaturing polyacrylamide gels with a 29:1 ratio of acrylamide: bisacrylamide followed by Gelred staining (Transgen Biotech, Beijing, China) and visualized by the GelDoc XR Molecular Imager System (Bio-Rad, USA). HRM primer pairs were designed according to resequence data of parents. HRM ran in the Eco™ Real-Time PCR System (Illumina), and the results were analyzed with Eco Software v3.0.16.0.

### RNA Isolation and Quantitative Real-Time PCR (RT-qPCR) Analysis

Total RNA of leaves was extracted as mentioned previously. Reverse transcription was performed with 1 μg of RNA through TransScript^®^One-Step gDNA Removal and cDNA Synthesis SuperMix (TransGen, Beijing, China) according to the manufacturer’s instructions. For the qPCR, 100 ng of total RNA were used for reaction in the Eco™ Real-Time PCR System (Illumina), and the expression levels were analyzed with Eco Software v3.0.16.0 and normalized with the results of soybean TUA5. PCR cycling conditions were set up with the following program: 94°C for 30 s, followed by 40 cycles of 94°C for 5 s, and 60°C for 30 s with fluorescence signal collection. Melting and cooling steps were set as default parameters. Relative gene expression levels were calculated using the 2^−ΔΔCt^ method. Three independent biological replicates were obtained and subjected to real-time PCR in triplicate. Raw data were standardized as described previously. All primers for expression analysis are listed in **Supplementary Table S1**.

For the diurnal expression pattern of *Glyma.12G073900* and *Glyma.U034500*, JLCLH and CN16 were grown at LD (16 light/8 dark) and then transferred to continuous light (LL) and dark conditions (DD), respectively. Leaves for RNA extraction were sampled at 3-h intervals at LD and 4-h intervals at DD and LL.

### Phylogenetic Analysis

Known PRRs family proteins were collected from the phytozome (https://phytozome.jgi.doe.gov/pz/portal.html) and NCBI (https://www.ncbi.nlm.nih.gov/). The phylogenetic tree was constructed using the neighbor-joining method (Saitou and Nei, 1987). The bootstrap consensus tree inferred from 1,000 replicates is taken to represent the evolutionary history of the taxa analyzed (Felsenstein, 1985). Branches corresponding to partitions reproduced in less than 50% bootstrap replicates are collapsed. In total, 46 nucleotide sequences including *Glyma.12G073900* and its homologues from soybeans and other plants were subjected to phylogenetic analysis. Evolutionary analyses were conducted in MEGA7 (Kumar et al., 2016).

## Result

### Phenotypic Performance for the Parents and the F_2_ Population

**Figure 1:**
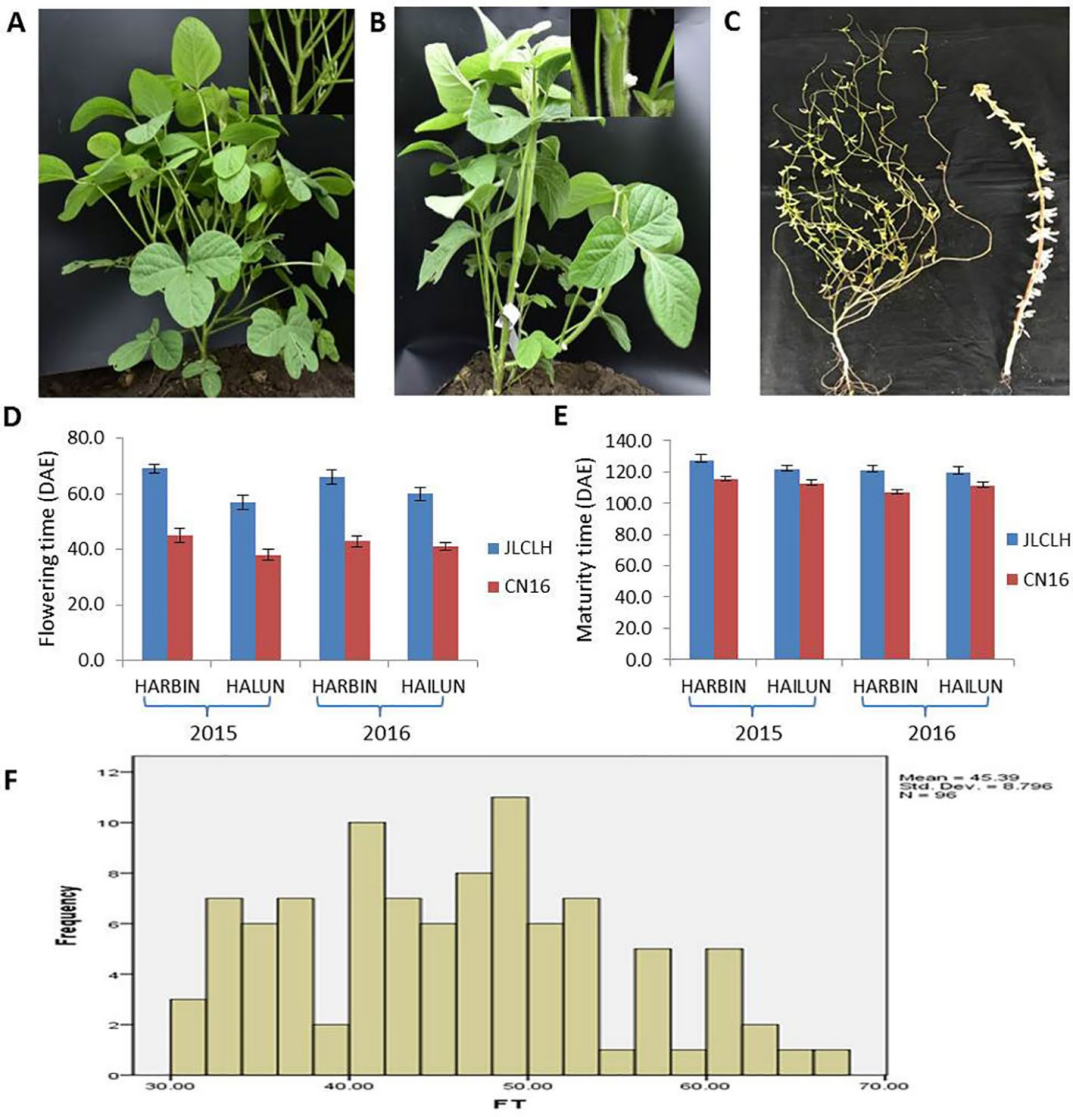
Phenotypic observation of the parents and the F_2_ population. **(A**, **B)** Plants of parents showing contrast difference in flowering time. When CN16 flowered, JLCLH was still in the vegetative growth stage. (A, JLCLH; B, CN16). **(C)** Plants showing contrast difference in maturity, CN16 (Right) reached R8 stage, whereas JLCLH (left) was at R5. **(D**, **E)** Graph shows R1 and R8 in 2015 and 2016 at HARBIN and HAILUN. **(F)** Frequency distribution of first flowering time (FT) in F_2_ population. F_2_ population (*n* = 96, at HAILUN in 2015) developed from a cross between JLCLH and CN16. DAE, days after emergence.

JLCLH, a landrace of semiwild soybean, has the same allelic genotypes of *E1e2E3HaE4* as CN16, an improved cultivar, at the *E1*, *E2*, *E3*, and *E4* loci (Zhai et al., 2014). However, JLCLH flowered on 65 (days after emergence, DAE), approximately 24 days later than CN16 (flowered on 41 DAE) at HARBIN location (**Figures 1A-C**). One-way ANOVA indicated that there were significantly difference (p < 0.01) in flowering time and maturity between two parents in both locations (**Figures 1D, E**). The flowering time in the F_2_ population exhibited a broad distribution with a range from 38 to 83 DAE (**Figure 1F**). It was hypothesized that there must be other locus or loci influencing flowering time besides *E1*, *E2*, *E3*, and *E4*.

### A Single Major QTL *qFT12-1* for Flowering Time Was Mapped to Chromosome 12

**Figure 2:**
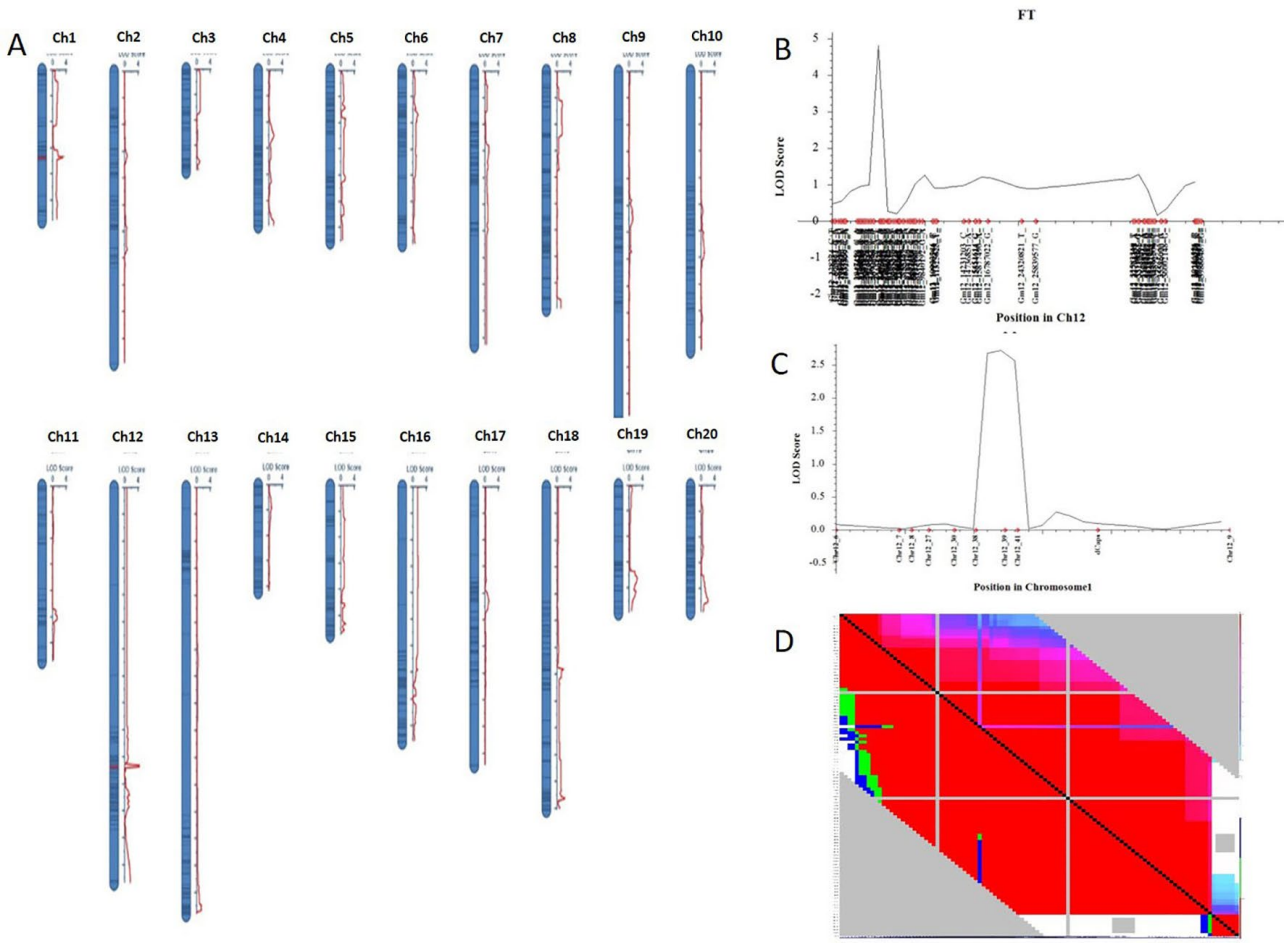
Genetic map of F_2_ populations with QTLs constructed with QTL IciMapping 4.0. **(A)** A set of 20 genetic linkages maps (corresponding to 20 chromosomes) with QTL. **(B)** QTL of chromosome 12 (*qFT12-1*). **(C)** Verification of *qFT12-1*. Chr, chromosome. **(D)** LD analysis using F_2_ population.

Nighty-six individuals of the F_2_ population were picked randomly and genotyped with Soy6K_MSU_SNP including 7,189 SNP markers (Akond et al., 2013; **Supplementary Table S4**). After exclusion of rare allele (a minor allele frequency of less than 10%) or unmapped markers, a total of 2,407 SNP markers were genetically mapped to 20 linkage groups corresponding to the 20 chromosomes of soybeans with QTL IciMapping 4.0 software (Meng et al., 2015) (**Figures 2A, B**; **Supplementary Table S5–7**). Basic descriptive statistics of the linkage maps is presented in **Table 1**. The map spanned 3,425.69 cm, with an average marker interval distance of 1.4 cm. The genetic length of each linkage groups ranged from 79.87 (Chr 3) to 293.23 cm (Chr 12). On average, each linkage group consists of 65 SNP markers, covering 171.28 cm. The recombination rates in the putative centromeric or pericentromeic regions were obviously suppressed in most chromosomes, as shown in the middle portions of each linkage group. In some other regions, gaps also exist due to lacking polymorphic markers between parents, indicating these regions are genetically homogenous between parents.

**Table 1:** Distribution of single nucleotide polymorphism (SNP) markers on the linkage map of the F_2_ population derived from JLCLH×CN16.

Group name	Number of markers	Length (cm)	Average marker density (cm)
Chr 1	86	115.94	1.35
Chr 2	107	218.18	2.04
Chr 3	100	79.87	0.80
Chr 4	128	119.04	0.93
Chr 5	124	130.66	1.05
Chr 6	117	132.51	1.13
Chr 7	196	205.05	1.05
Chr 8	256	179.2	0.70
Chr 9	94	256.04	2.72
Chr 10	69	209.48	3.04
Chr 11	49	133.99	2.73
Chr 12	100	293.23	2.93
Chr 13	185	315.67	1.71
Chr 14	62	83.47	1.35
Chr 15	86	114.71	1.33
Chr 16	159	191.97	1.21
Chr 17	119	208.74	1.75
Chr 18	155	240.78	1.55
Chr 19	62	98.92	1.60
Chr 20	153	98.24	0.64
Total	2,407	3,425.69	1.42

One QTL was identified for flowering time in the genetic linkage map constructed in the F_2_ mapping population with SNP markers. The major QTL *qFT12-1* was mapped on chromosome 12 with an LOD score of 4.5, PVE (phenotypic variance explained) of 20.5%, and DOM (dominance effect) of 2.7 (**Table 2**). In order to verify the authenticity of *qFT12-1*, we developed 10 polymorphic markers in the region of *qFT12-1*. When a total of 171 F_2_ plants were used, *qFT12-1* was confirmed (**Figure 2C**). Moreover, one of markers at nucleotide 5520945 showed significantly correlation with flowering time (*P* = 0.004), when we analyzed association of genotype and phenotype using 171 F_2_ population. We analyzed the two F_2_ populations grown in HARBIN (Population A) and HAILUN (Population B) respectively to test the effect of environmental factor on flowering time. Statistical analysis revealed that the environmental factor as well as interaction of environment × genetic significantly affect flowering time (**Table 3**). Although with a relative lower LOD, *qFT12-1* was detected in all environments, revealing *qFT12-1* is stable and true QTL. The relative lower LOD might reflect the complexity of flowering time gene network in which many other genes may also play minor or conditional roles in regulation of flowering time. The linkage disequilibrium (LD) was analyzed. The result showed that the LD block is rather large because of the LD (**Figure 2D**). It is difficult to narrow down the *qFT12-1*, so we used recombinants to further narrow down *qFT12-1*.

**Table 2:** The basic disruptive statistics of *qFT12-1* detected.

QTL	Physical position	Interval	LOD	PVE (%)	Additive effect	Dominance effect
*qFT12-1*	4671132-5238801	567.67 kB	4.5	20.5	5.5	2.7

**Table 3:** Statistical analysis of genetic effects of allelic variations of *qFT12-1* using dCAPS-X marker and the interactions on flowering time in two F_2_ populations in different environments.

Factor	df	Means square	*F*-value	*P*-value	Number	Location	Year
Population A	2	384.796	5.072	0.007**	171	HARBIN	2015
Population B	2	294.132	4.045	0.021*	96	HAILUN	2015
Environment	1	7,776.153	98.755	0.000***			
Genotype* Environment	2	629.05	8.416	0.000***			

*Statistical significance at 0.05 level; ** Statistical significance at 0.01 level.

### *qFT12-1* Was Delimited to a 56.4-Kb Region From 5496042 to 5552485 on Chromosome 12

To narrow down the genomic position of *qFT12-1*, the parents were resequenced at a higher coverage level to enhance the chances of developing more SNP and InDel markers. The average sequencing depth of the parents JLCLH and CN16 were 8.1× and 6.5×, respectively. The reference genome Gmax_275_Wm82.a2.v1 comprised 955,380,172 bp of assembled and anchored sequences. After data filtering, 2204598 in JLCLH and 1662895 in CN16 polymorphic SNPs and InDels were high quality, which could be used to design primers. Based on the resequencing data, 64 InDel primers were developed around and between the original map anchor markers Gm12_4670638_A_C and Gm12_5230528_G_A. PCR products amplified from 13 primers pairs showed clear polymorphisms between parents on agarose gel.

**Figure 3:**
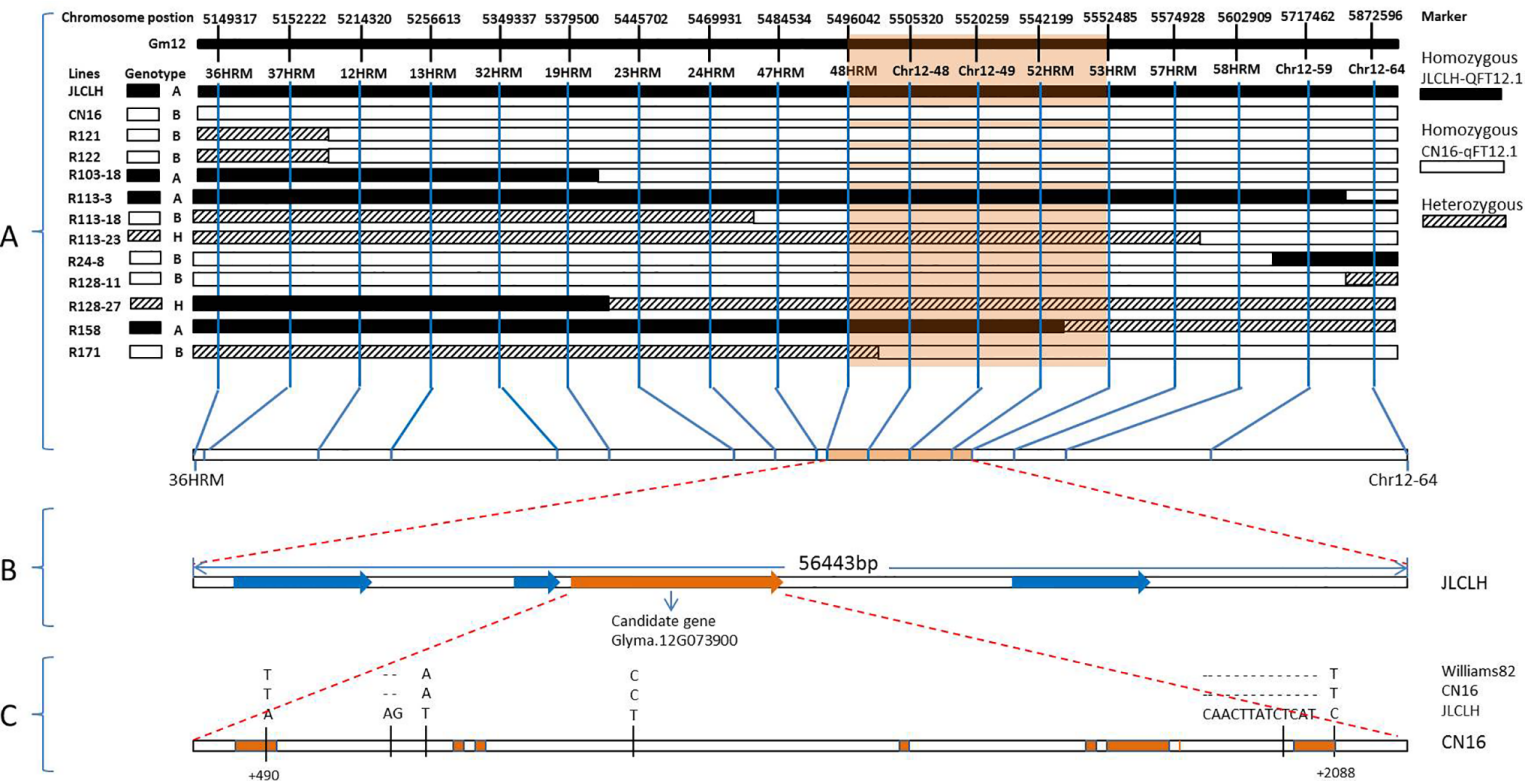
Positional cloning of the *qFT12-1*. **(A)** Graphical genotypes of 11 recombinants (R121, R122, R103-18, R113-3, R113-18, R113-23, R24-8, R128-11, R128-27, R158, and R171) carrying recombination events in the *qFT12-1* region. The genotypes of *qFT12-1* for these recombinants were confirmed based on the phenotypic segregation pattern in their progenies (**Supplementary Figure S1**). The delimited region for the *qFT12-1* is shaded in orange. **(B)** Within the delimited region in JLCLH, four genes (red) were identified, while *Glyma.12G073900* (orange) is the strong candidate gene. **(C)** Genetic variations of *Glyma.12G073900* among Williams 82, JLCLH, and CN16. The coding regions of *Glyma.12G073900* are marked in orange.

In general, the genotyping of 11 recombinants in the region of *qFT12-1* was performed for all 18 markers, and the results were indicated in **Figure 3**. The genotypes of *qF12-1* were reflected from phenotypes of progenies of these recombinants, i.e., early flowering homozygous CN16 (*qFT12-1*), later flowering homozygous JLCLH, and heterozygous JLCLH/CN16 genotypes. Early flowering phenotypes for the progeny of R121, R121, R103-18, in combination of heterozygous phenotype for R128-27 revealed that the *qFT12-1* exist on right side of 24HRM. The late flowering time for progeny of R113-3, early flowering times for progeny of R24-8 and R128-11, and in combination of heterozygous phenotype for R113-23 can delimit *qFT12-1* to the left side of 58HRM. Furthermore, the R158 and R171 could narrow down the *qFT12-1* region further. From **Figure 3**, the genotypes of *qFT12-1* of R158 and R171 are homozygous JLCLH and homozygous CN16 type judging from the phenotype of progenies of two recombinants, we can delimit the *qFT12-1* to a 56.4-kB region between markers of 48HRM and 53 HRM. Furthermore, heterozygous recombinants R113-23 and R128-27 were used to test association between genotype of markers and phenotype in progeny. We genotyped progenies of R113-23(n = 31) and R128-27(n = 32) using 52HRM marker. Homozygous JLCLH and homozygous CN16 genotypes of 52HRM showed significant correlation in flowering time in progenies of recombinants R113-23 and R128-27 (*P* < 0.03 and *P* < 0.01, respectively) (**Table 4**). Taken together, *qFT12-1* was narrowed down to the 56.4-kB region between 48HRM and 53HRM. According to the Williams 82.a2.v1 genome annotation, four genes were annotated: three annotated genes (*Glyma.12G073700*, *Glyma.12G073900*, and *Glyma.12G074000*) having putative functions and *Glyma.12G073800* with unknown function (**Table 5**).

**Table 4:** Associated segregation of genotypes and phenotype of flowering time in recombinants in field conditions. AA represents JLCLH allele, BB represents CN16 allele, AB represents heterozygous.

Family	No. of plants	Flowering time (mean ± SD)			One-way ANOVA	
		AA	AB	BB	F-value	Probability
F_3_						
R121	59			41.9 ± 4.2		
R122	39			44.5 ± 4.9		
R158	40	65.6 ± 4.5				
F_4_						
R103-18	36	64.2 ± 2.3				
R113-3	36	63.0 ± 4.1				
R113-18	45			47.9 ± 6.9		
R113-23	31	63.3 ± 7.3	54.9 ± 7.7	45.0 ± 2.5	7.1	0.003
R24-8	27			45.3 ± 3.3		
R128-11	48			45.9 ± 4.2		
R128-27	32	60.8 ± 6.1	54.9 ± 5.4	43.0 ± 3.5	8.9	0.001
R171	38			43.2 ± 3.6		

**Table 5:** Functional annotation for the delimited *qFT12-1* region.

Soybean gene id	Gene start	Gene Stop	Description	Pathway
*Glyma.12G073700*	5497564	5501763	Mitogen activated protein kinase	
*Glyma.12G073800*	5507147	5508540	unknown function	–
*Glyma.12G073900*	5508365	5522772	pseudo-response regulator 7	phosphorelay signal transduction system
*Glyma.12G074000*	5534233	5538664	Protein kinase superfamily protein	Interleukin-1 receptor-associated kinase 4

### *Glyma.12G073900* Is the Strongest Candidate Gene

**Figure 4:**
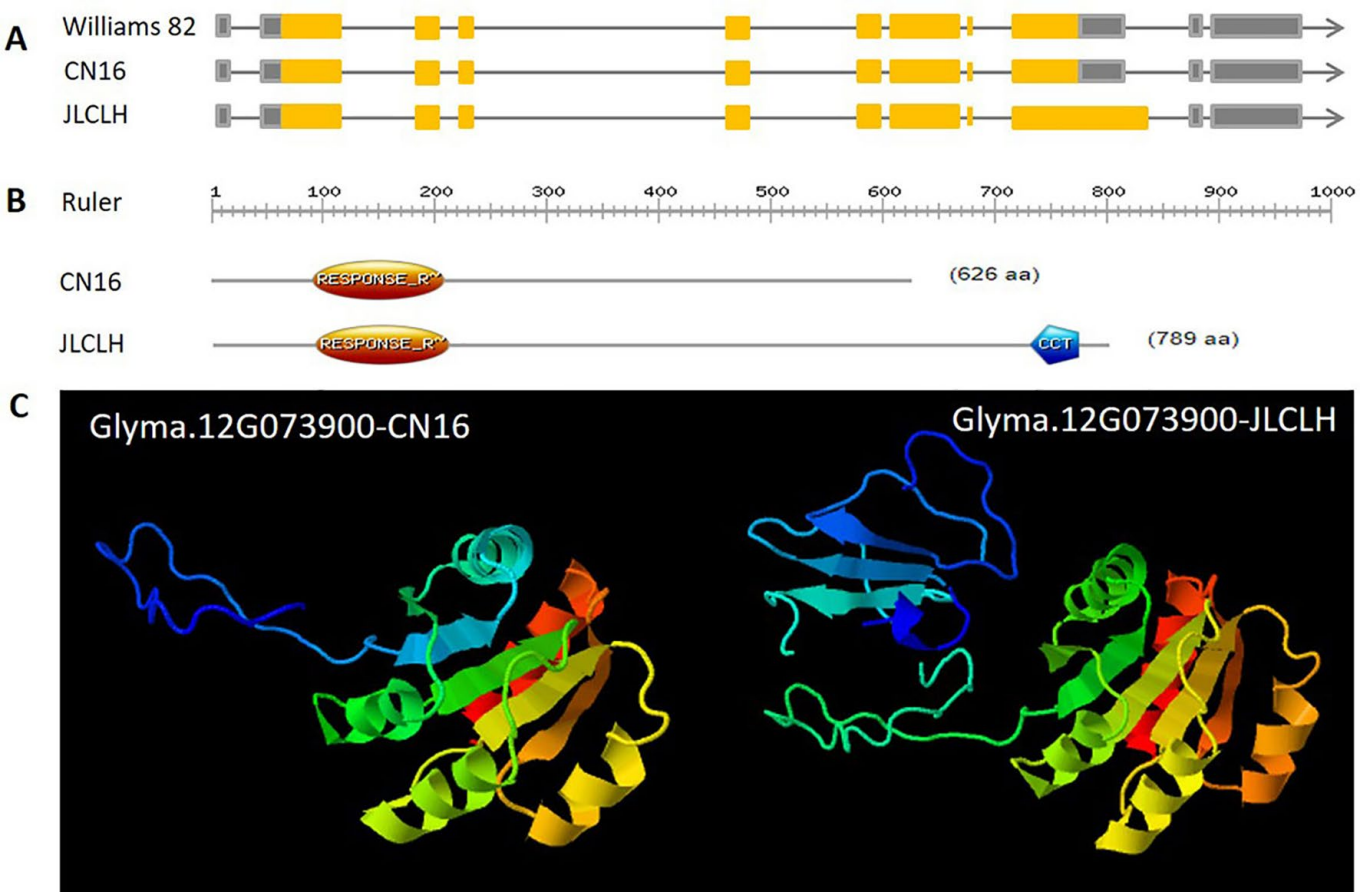
Genetic, protein structure and putative 3D protein structures of *Glyma.12G073900*. **(A)** Genetic structure of *Glyma.12G073900* in CN16 and JLCLH. **(B)** Protein structure of *Glyma.12G073900* in CN16 and JLCLH. Structure analyzed using online software (http://prosite.expasy.org). **(C)** Putative 3D protein structures of *Glyma.12G073900* in CN16 and JLCLH. Structure prediction was conducted online (http://www.sbg.bio.ic.ac.uk/phyre2/html/page.cgi?id = index).

When four candidate genes were compared between parents using the resequenced data, there are three SNPs in introns of *Glyma.12G073700*, one SNP in intron of *Glyma.12G073800* and no polymorphic SNP in *Glyma.12G074000*. However, a total of six SNPs, four in intron and two in exon, have been detected in *Glyma.12G073900* (**Table 6**). *Glyma.12G073900* is predicted to encode pseudo-response regulator (PRR) protein with a pseudo receiver domain at the N terminus and a CCT motif at the C terminus (**Figure 4B**). PRR genes are clock-associated protein factors known to be key regulator in photoperiod network in many grains (Murakami et al., 2005; Nakamichi et al., 2010). Among two SPNs occurred in exons of *Glyma.12G073900*, the first one occurred at the first exon, at nucleotide 490, leading to A in JLCLH to C in CN16. This nonsynonymous mutation caused a change glutamine (Gln) in JLCLH to leucine (Leu) in CN16 at amino acid level. The second occurred at nucleotide 2088, this mutation leading to C in JLCLH to T in CN16, resulting in a premature termination codon (PTC) occurred in CN16 (**Figure 4A**). We predicted gene interaction on Soynet (https://www.inetbio.org/soynet/), a functional gene network and coexpression network database for a legume crop, soybean (*Glycine max*). There are mutual regulatory relationships between *Glyma.12G073900* and *E2*, *E3*, *E4*, and *GmFTs*. *Glyma.12G073900* and its homologous *Glyma.U034500* are interrelated with *E2* directly (**Supplementary Figure S2**). Three-dimensional (3D) structures of supposed PRR proteins from JLCLH and CN16 were predicted. As expected, the putative 3D protein structures of coding protein between parents were different at the C terminus. Putative PRR protein containing a CCT motif in JLCLH has four additional beta sheets at C terminus (**Figure 4C**) when compared with that in CN16. Therefore, we asserted that *Glyma.12G073900* is a candidate gene for *qFT12-1* regulating flowering.

**Table 6:** Allelism of four candidate genes between JLCLH and CN16 revealed through whole-genome resequencing.

Gene ID	Position	JLCLH	CN16	Refference	Polymorphism type	Polymorphism position	Effect
Glyma.12G073700	–	A	G	G	SNP	INTRON	
	–	G	G	A	SNP	INTRON	
	–	C	C	T	SNP	SPLICE_SITE	
Glyma.12G073800	–	A	G	G	SNP	INTRON	
Glyma.12G073900	490	A	T	T	SNP	EXON	NSC CAG[Q]- CTG[L]
	–		AG	AG	InDel	INTRON	
	–	T	A	A	SNP	INTRON	
	–	T	A	A	SNP	INTRON	
	–	CAACTTATCTCAT	–	–	InDel	INTRON	
	2088	C	T	T	SNP	EXON	NSC CAA[L]- TAA[*]

NSC, Non Synonymous coding.

**Figure 5:**
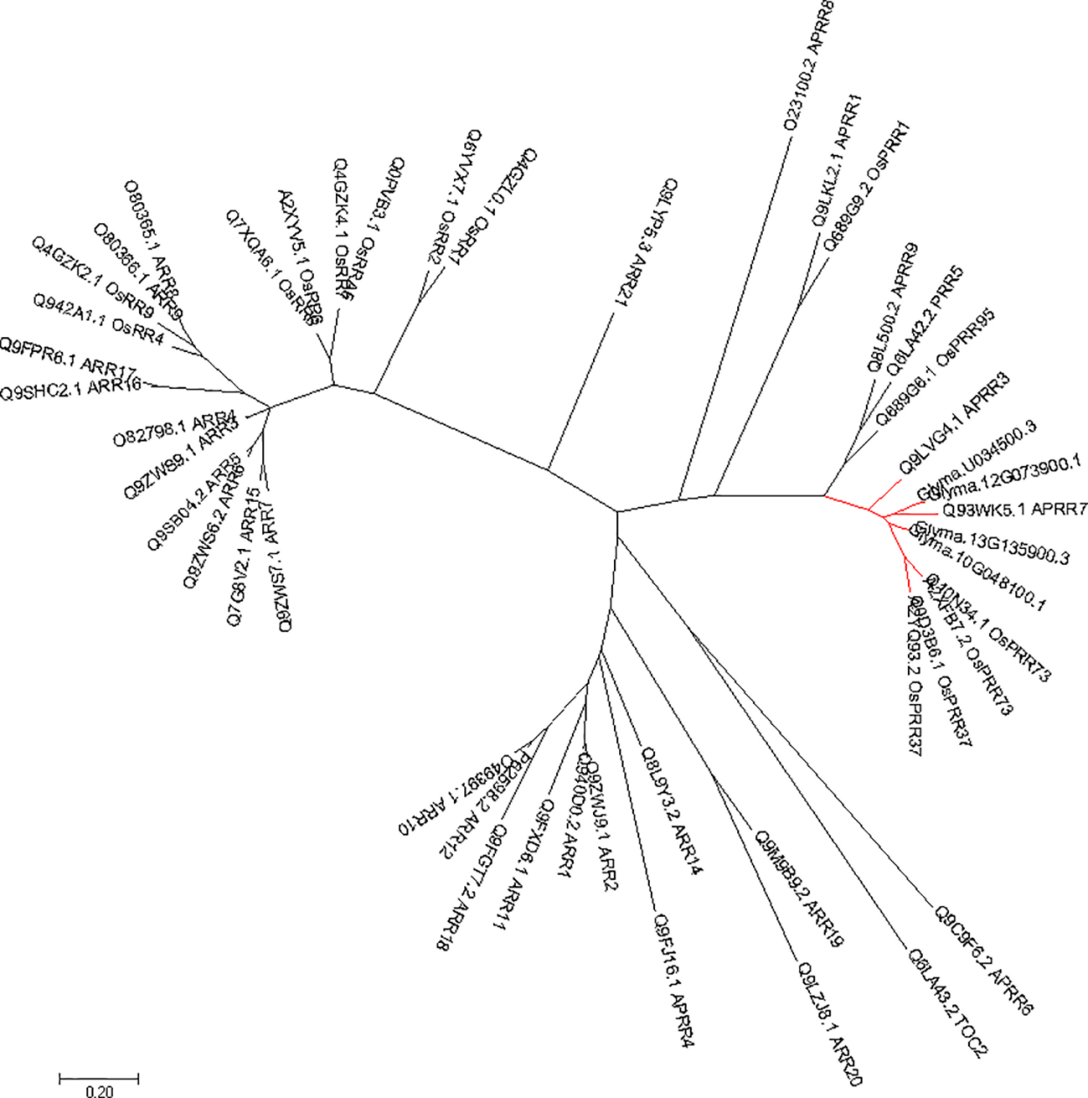
Phylogenetic analysis of *Glyma.12G073900* in soybeans and other species. The gene names and accession numbers were retrieved from NCBI (https://www.ncbi.nlm.nih.gov/). Accessions numbers are list in **Supplementary Table S2**.

**Figure 6:**
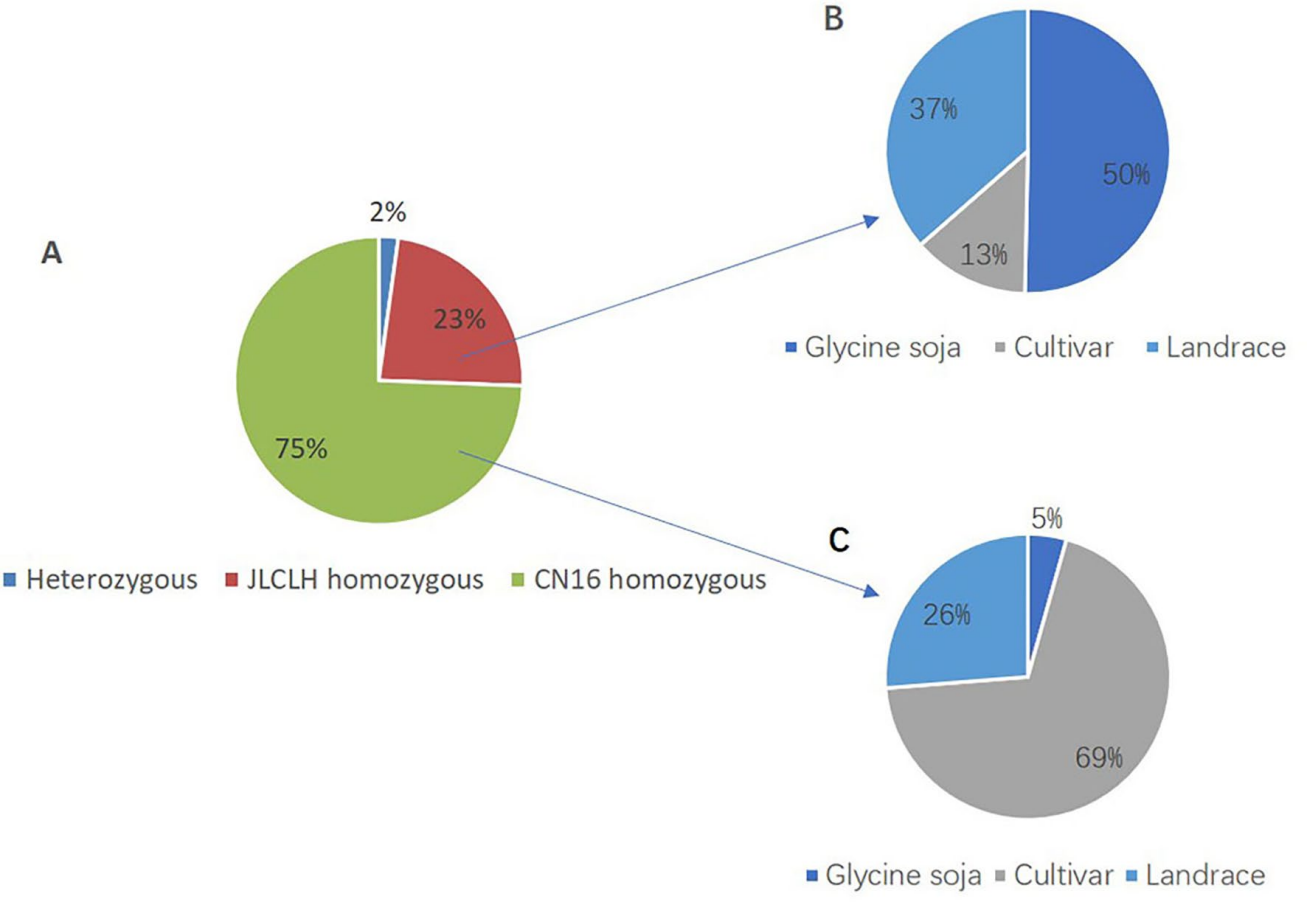
SNP polymorphisms percentage at nucleotide 2088 of *Glyma.12G073900* in 642 soybean accessions. **(A)** Percentage of three SNP types (JLCLH homozygous, CN16 homozygous, and heterozygous) at nucleotide 2088 of *Glyma.12G073900* in 642 soybean accessions. **(B)** Percentage of JLCLH homozygous type in *Glycine soja* accessions, cultivars, and Landraces. **(C)** Percentage of CN16 homozygous type in *Glycine soja* accessions, cultivars, and landraces.

Phylogenetic tree of *PRRs* identified in Arabidopsis, rice, indicates that *PRR* genes *Glyma.12G073900* and *Glyma.U034500* belong to the same clade (**Figure 5**) as *PRR3*, *PRR7* from Arabidopsis. When we compared genetic variations of *Glyma.12G073900* in 642 soybean accessions (**Supplementary Table S3**), 75% of cultivars or accessions are of CN16 genotype (at nucleotide 2088), whereas 23% are of JLCLH genotype (**Figure 6**). Among 151 cultivar or accession carrying JLCLH genotype, 76 accessions are from *Glycine soja*, 55 accessions are landraces, and 20 accessions are modern cultivar (**Table 7**). In contrast, among 480 CN16-type accessions, 333 accessions are improved cultivars, 126 accessions are landraces, and 21 accessions are from *Glycine soja* (**Table 7**). The result indicated JLCLH allelic type widely exists in *Glycine soja* while CN16 allelic type is wide distributed in improved cultivars, especially from Northern part of China. 182 of 333 accessions have maturation data, we found 149 accessions are from maturity group 000 to IV, in contrast, only 33 accessions are from maturity group V to X.

**Table 7:** SNP polymorphisms type at nucleotide 2088 of *Glyma.12G073900* in 642 soybean accessions.

SNP Type	Total number	Glycine soja	Cultivar	Landrace
Heterozygote	11	11	–	–
JLCLH Homozygous	151	76	20	55
CN16 Homozygous	480	21	333	126
Total	642	108	353	181

### Diurnal Circadian Expression Pattern of *Glyma.12G073900* and *Glyma.U034500*

**Figure 7:**
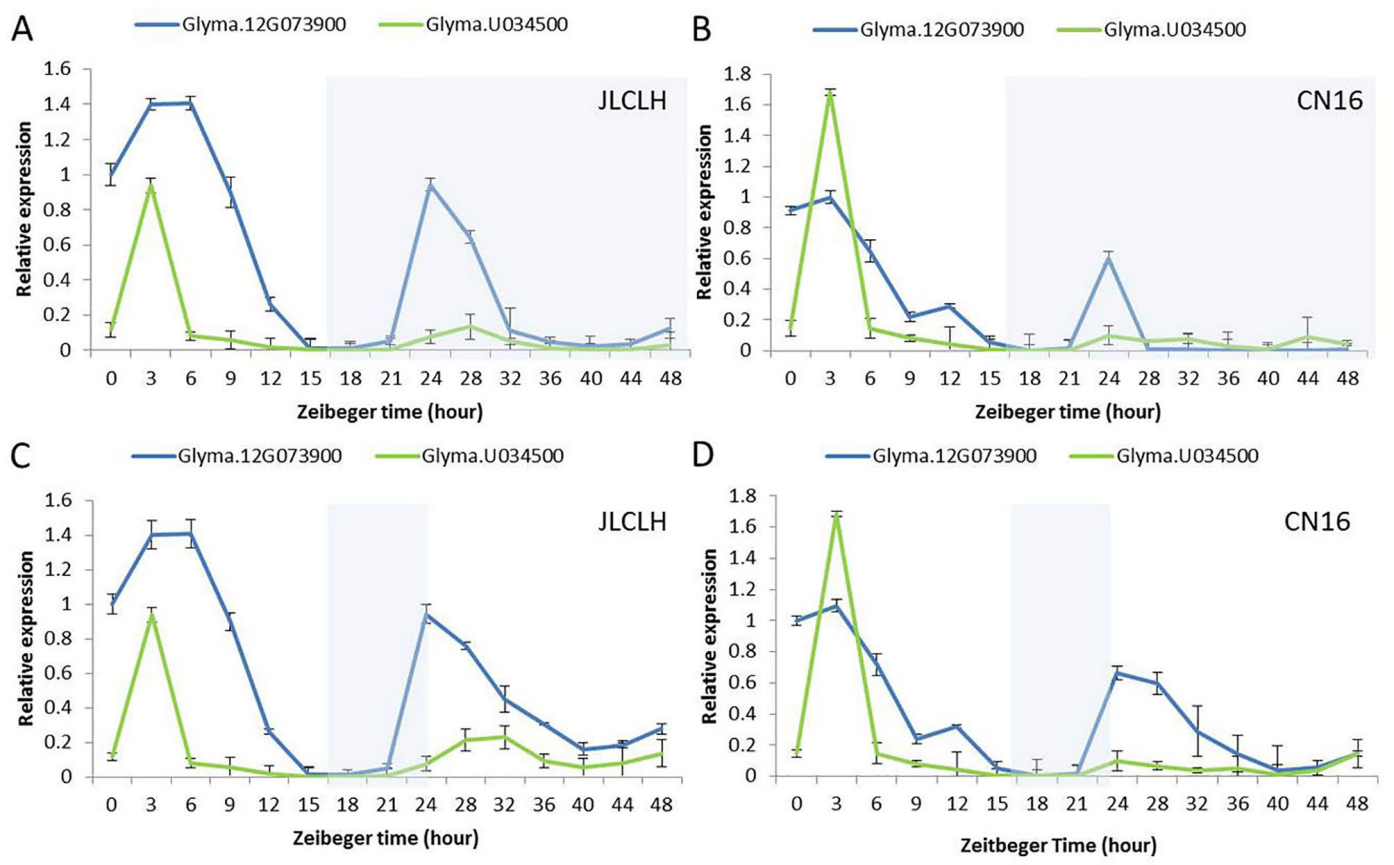
Diurnal expression pattern of *Glyma.12G073900* and *Glyma.U034500* in parents (JLCLH and CN16). **(A**–**B)** Diurnal expression of *Glyma.12G073900*, *Glyma.U034500* in JLCLH and CN16 respectively at LD-LL condition [plants were grown under 16-h light/8-h dark (LD) cycle for 20-d-old seedlings and then released to continuous light (LL)]. **(C**–**D)** Diurnal expression of *Glyma.12G073900*, *Glyma.U034500* in JLCLH and CN16 respectively at LD-DD condition [plants were grown under 16-h light/8-h dark (LD) cycle for 20-d-old seedlings and then released to continuous dark conditions (DD)]. RNA samples were prepared from leaves at 3 to 4 h intervals. Data shown are relative to the control gene *TUA5* and represent means ± SD for three biological replicates. The shaded region indicates the dark period.

The diurnal expression pattern of *Glyma.12G073900* and its homologue in soybeans may help us to clarify whether the candidate gene regulates flowering time through the circadian clock related pathway. Under LD (16 h light/8 h dark), *Glyma.12G073900* showed diurnal expression in both JLCLH and CN16, and in both LL and DD condition (**Figures 7A–D**), the expression peaks were around ZT3 to ZT6 in the parents. *Glyma.U034500*, the most homologous gene to *Glyma.12G073900*, showed a similar diurnal expression rhythm, but its expressional abundance is lower than *Glyma.12G073900* under LD, and significantly lower under LL and DD.

## Discussion

### Genes or QTLs Involved in Regulation of Flowering Time Trait in Soybeans

Flowering time, a vital agronomic trait, influences the productivity, adaptability, and quality of soybeans. Understanding the genetic mechanism of flowering time and photoperiod insensitivity is advantageous for promoting final yield in specific regions. To date, six major *E* genes for maturity of soybeans (*E1*, *E2*, *E3*, *E4*, *E9*, and *E10*) have been identified. In order to locate novel loci or genes of flowering time in soybeans, parents of the mapping population used in this study were chosen because they had large differences in flowering time although having the same allelic genotypes at *E1* to *E4*. We detected and characterized a major flowering time QTL, *qFT12-1*, on chromosome 12 in soybeans. Of the 104 flowering time QTLs archived in SoyBase (https://soybase.org/sbt/), one QTL has been reported on chromosome 12 with additive effect of −1.8 to −3.2 days, 26.6–36.8% of PVE at the 19.32-cm interval between Satt568 and Satt442 (Kuroda et al., 2013). Removing the effect of *E1*, a QTL *qFT-H* on chromosome 12 with LOD 2.38 with nearest marker of Satt442 in 28.2-cm interval was detected (Lu et al., 2016). Kuroda et al. (2013) identified the flowering time QTL including the region of *qFT12-1* by using F_2_ populations derived from crosses between a wild soybean accession (JP110755) and an improved soybean cultivar, Fukuyutaka. Liu et al. (2018) mapped *qFT12.1* to a 2703-kB (44.04 cm) interval between the markers BARCSOYSSR_12_0220 and BARCSOYSSR_12_0368 on chromosome 12 with an LOD score of 15.16 and PVE of 38.27% using a CSSL population derived from a soybean cultivar, Jackson (PI548657) and a wild soybean accession, JWS156-1. The existence of QTL for flowering time frequently detected in different populations and environments suggests that a key gene in this region on chromosome 12 plays important role in controlling flowering time. Besides flowering time QTLs, two more pod maturity QTLs were detected around this region. Moreover, a genome-wide association study (GWAS) showed that seven more flowering time QTLs on chromosome 12 (Zhang et al., 2015).

### *qFT12-1* Region Has Been Subjected to Extensive Selection During Evolution and Domestication

Modern cultivated soybean was widely believed to domesticate from wild soybean (*Glycine soja*) (Kim et al., 2012). It has been proven that wild soybean or landraces preserve higher genetic diversity including genes controlling flowering time, providing genetic resource pools for the improvement of cultivated soybeans (Zhou et al., 2015). Domestication could increase plant adaptability to different environments (Allaby, 2010). In sorghum, there are many loss-of-function alleles encoding truncated PRR37 protein. Each *PRR37* allelic variant could be traced to specific geographic location or specialized agronomic type (Klein et al., 2015), indicative of human selection. In this study, when we analyzed the variation of *Glyma.12G073900* at nucleotide 2088 in 642 soybean accessions, it was found that JLCLH homologous type mostly exists in wild soybeans and landrace, whereas CN homozygous type are mostly distributed in improved cultivar. An inDel located on 5520259 (reference genome position) in *Glyma.12G073900* has been identified, which can clearly distinguish wild soybeans from modern soybean cultivars when whole genome resequencing of another batch of soybean germplasms were analyzed (Song QJ, personal communication). This result reassure that this gene is related to domestication and the region is within domestication sweep. Among 182 soybean accessions or cultivars being assigned to the maturity groups (**Supplementary Table S3**), 149 accessions or cultivars were belonged to MG 000 to MG IV suitable for cultivation in high latitude. The allelic type *Glyma.12G073900* in JLCLH is probably ancestral genotype and this gene has been subjected to natural selection in evolution and artificial selection during domestication process. According to the analysis of resequencing data of 302 wild and cultivated accession of soybean, the region of chromosome12: 5271201-58199864 is within domestication sweep, in the proximal region of *qFT12-1*, a gene controlling pubescence has been subjected to intensively artificial selection. The presence of *qFT12-1* have been reported by different researchers using different populations all of which were derived wild soybean × improved cultivars (Kuroda et al., 2013; Liu et al., 2018). We assumed that CN16 homozygous genotype of *qFT12-1* is generally required for modern cultivars to reduce photoperiod sensitivity to adapt to higher latitudes.

### *PPR* Homolog May Be Involved in Circadian Clock and Flowering Time Regulation in Soybean

PRRs are key components of transcription/translation circadian networks in plants (Farré and Liu, 2013). PRRs are core genes in the translational feedback loop mechanism of the central oscillator in *Arabidopsis* (Hayama and Coupland, 2003; Nakamichi et al., 2007). *PRR9*, *PRR7*, and *PRR5* of Arabidopsis were identified to suppress *CCA1* and *LHY* promoter activities (Nakamichi et al., 2010). PRR proteins with a pseudo receiver domain at the N terminus and a CCT motif at the C terminus were generally proposed to contribute to day-length measurement through control of the time-keeping mechanism associated with *CO* transcription (Yamamoto et al., 2003; Murakami et al., 2004). *OsPRR37* was mapped very closely to *Hd2*-QTL, which was identified as the major locus that enhances the photoperiod sensitivity of flowering (Murakami et al., 2005). In sorghum accession ATx623, one additional nucleotide substitution in *SbPRR37* resulted in a truncated protein without CCT motif, which leaded to photoperiod insensitive and early flowering (Murphy et al., 2011). Expression of floral repressors *SbPRR37* flowering under LD conditions inhibits flowering in sorghum (Yang et al., 2014). In this study, the candidate gene for *qFT12-1*, *Glyma.12G073900* is the homogenous to *PRR7* of Arabidopsis. A single mutation occurred at the end of the eighth exon, *Glyma.12G073900* in CN16 resulting in a CCT motif truncated protein. CCT motif might be the key domain for its function in regulation of flowering. Circadian rhythms in expression of *Glyma.12G073900* both in JLCLH and CN16 are well accordance with that PRR domains in *Glyma.12G073900* are conserved in JLCLH and CN16. As short day plant, soybean has a special gene network for regulation of flowering which is different from Arabidopsis. In soybean, *E1* is the most important gene in flowering regulation network. In flowering pathway of soybean, *E1* accepts light signal from phytochrome through some circadian clock genes, regulates flowering by expression of *GmFTs* (*GmFT2a*, *GmFT5a*, *GmFT4*). In this study, the candidate gene of *qFT12-1, Glyma.12G073900* has characteristic of circadian clock gene and interactively interoperate with *GmFTs* as showed prediction network. If it had, *Glyma.12G073900* most probably conducts its function with *E2* encoding GIGANTEA. More research is needed to verify this hypothesis and how *qFT12-1* coordinately controls flowering time in cooperation with the *E1* pathway.

## Conclusion

In the present study, a major QTL for flowering time, *qFT12-1*, was identified, validated, and characterized. At first, we mapped the *qFT12-1* to an approximately 567-kB region on chromosome 12. Using recombinants whose recombination events were occurring within the QTL region allowed us to narrow down the QTL region to 56.4 kB. The *PRR7* homolog *Glyma.12G073900* is proven to be the strongest candidate gene for *qFT12-1*. Further studies are needed to determine the effect of *qFT12-1* in other genetic backgrounds, as well as its interaction with other flowering time genes and with environmental conditions, such as temperature and light quality. Regardless, the deciphering of molecular basis of *qFT12-1*, a QTL of flowering time will not only contribute to a better understanding of the molecular basis of the photoperiod-dependent regulation of flowering time but could also be potentially useful in improving the adaptability and productivity of cultivated soybeans.

Raw data of SoySNP8k iSelect BeadChip can be download at. 10.6084/m9.figshare.9857312.

Raw data of resequencing of JLCLH and CN16 can be download at 10.6084/m9.figshare.9863636.

## Data Availability Statement

Publicly available datasets were analyzed in this study. These data can be found here: https://figshare.com/articles/Raw_data_of_chip_for_QTL_IciMapping_txt/9857312

## Author Contributions

ZX conceived this project. YL and YD performed most of the experiments in the laboratory. HW, BH, HZ, and JY conducted field experiment and phenotypic observation. ZX and YL wrote the article. All authors reviewed the final manuscript.

## Funding

This work was supported by the National Key R&D Program of China (2016YFD0100201 and 2016YFD0101900), by Programs (31471518, 31771869, 31771818, 31901566) from the National Natural Science Foundation of China, by program (CXGC2018JC003) from the Agricultural Science and Technology Innovation Project of Jilin Province of China, by the Regional Key Project of Science and Technology Service (KFJ-STS-QYZD-037), and by the Key Deployment Projects (ZDRW-ZS-2019-2) of the Chinese Academy of Sciences.

## Conflict of Interest

The authors declare that the research was conducted in the absence of any commercial or financial relationships that could be construed as a potential conflict of interest.
