# Supplementary figures and images for: Positional Cloning of the Flowering Time QTL qFT12-1 Reveals the Link Between the Clock Related PRR Homolog With Photoperiodic Response in Soybeans
Source: Front Plant Sci. 2019 Oct 15;10:1303. doi: 10.3389/fpls.2019.01303 (PMC6803524; doi:10.3389/fpls.2019.01303)

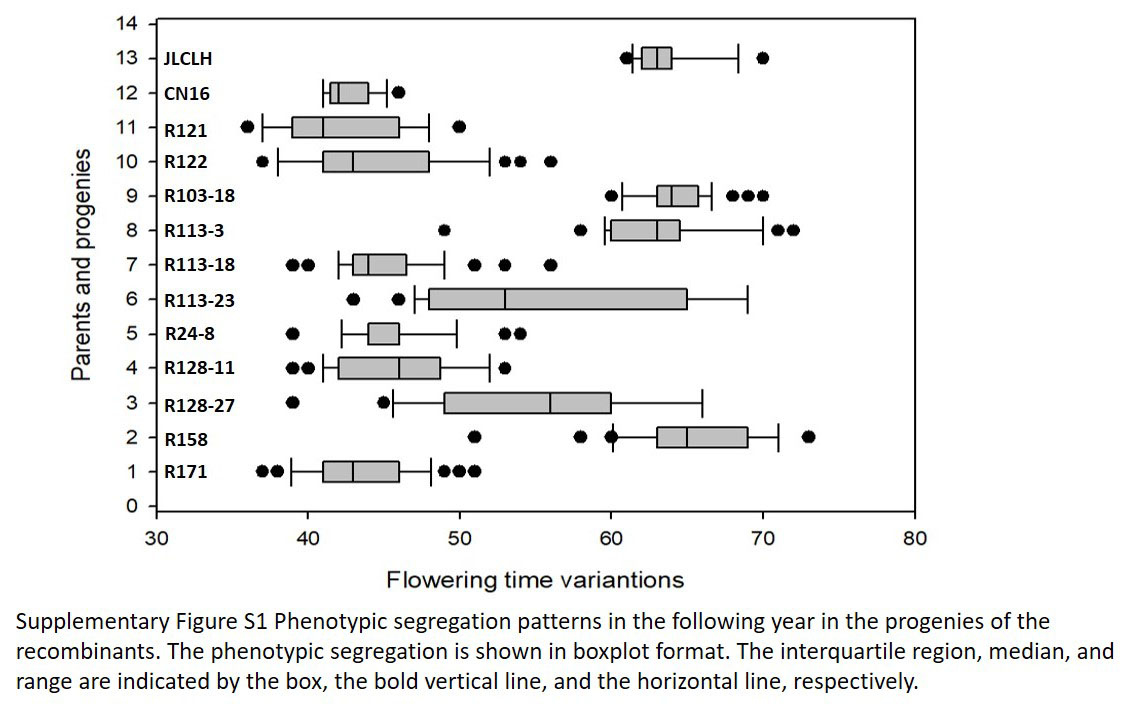

Supplement: Supplementary file 1 [file Image_1.jpg]

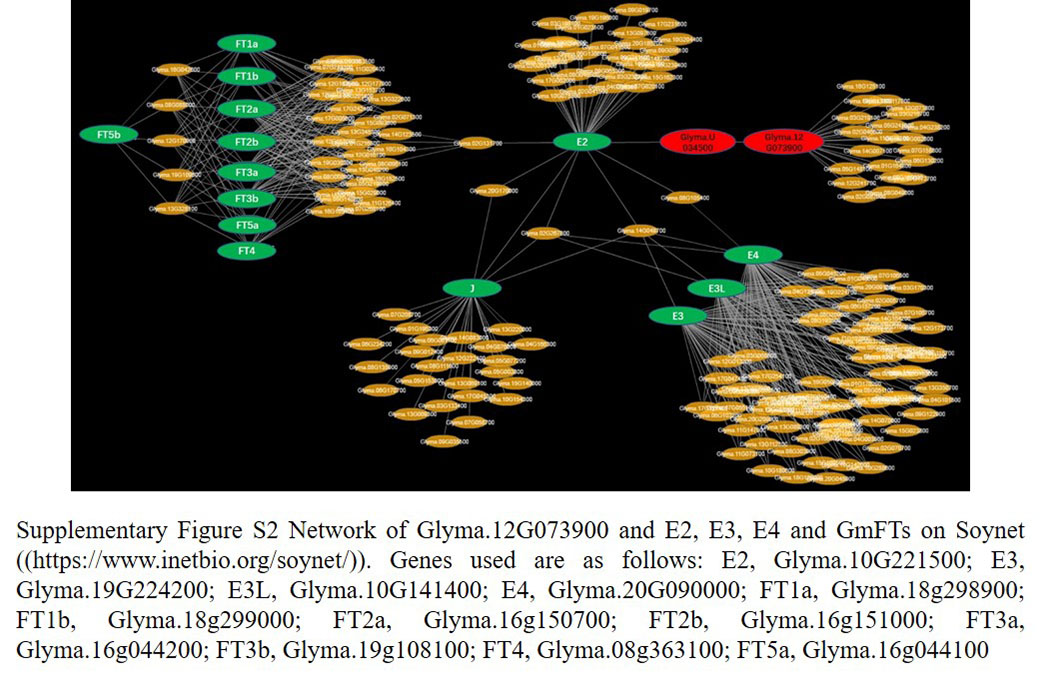

Supplement: Supplementary file 2 [file Image_2.jpg]
