# Supplementary material for: Positional Cloning of the Flowering Time QTL qFT12-1 Reveals the Link Between the Clock Related PRR Homolog With Photoperiodic Response in Soybeans
Source: Front Plant Sci. 2019 Oct 15;10:1303. doi: 10.3389/fpls.2019.01303 (PMC6803524; doi:10.3389/fpls.2019.01303)
Supplement: Supplementary file 3 [file Table_1.docx]

Supplementary Table S1 Primers used in this study

| Primer name | Primer sequence 5' to 3' | Note |
| --- | --- | --- |
| 12HRM-F | TGCTTCAACTTTATCACTTCCCA | For genotyping |
| 12HRM-R | TGCTATTGGGATTGATGAGGG | For genotyping |
| 13HRM-F | TTTACCAATCGTAGTCAACTCA | For genotyping |
| 13HRM-R | TGAGCTTTGAAATCTCTCGATCA | For genotyping |
| 19HRM-F | TGAGCTTTGAAATCTCTCGATCA | For genotyping |
| 19HRM-R | CTTCTGCGGTGTGTTCCAAT | For genotyping |
| 23HRM-F | ATCTCGGACAATGTACAACC | For genotyping |
| 23HRM-R | GGTATCTCAGGAACATCAACTC | For genotyping |
| 24HRM-F | TAGAGTCATGGCTGGAGGTA | For genotyping |
| 24HRM-R | GACAAGCAATACTGAGTTCTCA | For genotyping |
| 32HRM-F | AACTGCGTGGAGCTTTATCC | For genotyping |
| 32HRM-R | CCCACTTCCCATCAAATTAGA | For genotyping |
| 36HRM-F | GATGCCTCCCCAGTGATTG | For genotyping |
| 36HRM-R | TCAACTCAGTCGAAAAGGGT | For genotyping |
| 37HRM-F | AAACACGTGCACATCAGTAG | For genotyping |
| 37HRM-R | TGAAAGTTATCAGCTTGCGA | For genotyping |
| 47HRM-F | TCCAAACAATCTTTATGCGTT | For genotyping |
| 47HRM-R | GATTTCCAGGAGTGTCACTAC | For genotyping |
| 48HRM-F | GCTCATCAAGTTCATCATTCCA | For genotyping |
| 48HRM-R | TCTCAGTTTTATGGGAAAAGGT | For genotyping |
| 52HRM-F | AGCCGAAGTTGACATCTTTT | For genotyping |
| 52HRM-R | GTGCCATATGTCCTGCTTTT | For genotyping |
| 53HRM-F | GGGAAGAAATTGCAGGTGTG | For genotyping |
| 53HRM-R | GAAGCACCTATTTACAATACTCA | For genotyping |
| 57HRM-F | TGATTTTGAACAACCAACCCA | For genotyping |
| 57HRM-R | TCTGTATCAGCCAGCTTTCT | For genotyping |
| 58HRM-F | TTGTTGGAGTTGATGGAATTATT | For genotyping |
| 58HRM-R | GCAATTGCGTCCATTCATTTC | For genotyping |
| Chr12-48-F | CCCAATTCTTAAGGCTAACG | For genotyping |
| Chr12-48-R | GGATTGATTGATTCTCAACC | For genotyping |
| Chr12-49-F | TGATGCTCTTCAGTCCTTTG | For genotyping |
| Chr12-49-R | ACATTTCCAGTTTGAGCCTG | For genotyping |
| Chr12-59-F | CAGGGCATGATCATCACCAA | For genotyping |
| Chr12-59-R | TGAGGGAGTAAATGATTGGGA | For genotyping |
| Chr12-64-F | ACTCGTGTTGAAGGTGTGTC | For genotyping |
| Chr12-64-R | CCTCCTCCTAGCCAAAGTCT | For genotyping |
| dCAPS-X-F | CTGCAGTAGATCCCCAATCAGATCATGA | For genotyping |
| dCAPS-X-R | CTGCAGTAGATCCCTAGTCAGATCATGA | For genotyping |
| TUA5-F | TGCCACCATCAAGACTAA | For real-time PCR |
| TUA5-R | ACCACCAGGAACAACA | For real-time PCR |
| 12G073900-F | TTTGACAAGAAGCCGGAGTC | For real-time PCR |
| 12G073900-R | TTTGACAAGAAGCCGGAGTC | For real-time PCR |
| E1-F | CACTCAAATTAAGCCCTTTCA | For real-time PCR |
| E1-R | TTCATCTCCTCTTCATTTTTGTTG | For real-time PCR |
| GmFT2a-F | ATCCCGATGCACCTAGCCCA | For real-time PCR |
| GmFT2a-R | ACACCAAACGATGAATCCCCA | For real-time PCR |
| GmFT5a-F | AGCCCGAACCCTTCAGTAGGGA | For real-time PCR |
| GmFT5a-R | GGTGATGACAGTGTCTCTGCCCA | For real-time PCR |
